# Re-irradiation for recurrent esophageal cancer: clinical benefit, survival outcomes, and toxicity profile

**DOI:** 10.1016/j.ctro.2025.101068

**Published:** 2025-11-03

**Authors:** Chiara Mattioli, Lucy A. van Werkhoven, M. Loi, Joost J. Nuyttens

**Affiliations:** a Radiation Oncology, Azienda Ospedaliero Universitaria Careggi, Università di Firenze, Firenze, Italy; b Department of Radiotherapy, Erasmus MC Cancer Institute, University Medical Center Rotterdam, the Netherlands

## Abstract

**Background and purpose:**

To evaluate survival and toxicity in patients with recurrent esophageal cancer treated with curative (cRT) and palliative (pRT) reirradiation.

**Materials and methods:**

From May 2015 to August 2024, 41 patients with locally recurrent esophageal cancer received cRT or pRT. Toxicity was assessed using CTCAE v5.0. Overall survival (OS) was analyzed using the Kaplan-Meier method. Clinical benefit of pRT (reduction in dysphagia, pain or bleeding) was assessed 4 weeks after reRT.

**Results:**

The cohort consisted of 41 patients (median age 73 years). At reirradiation, 36 patients received pRT (20 Gy/5fx or 30 Gy/10fx), and 5 received cRT (50.4 Gy/28fx with chemotherapy). Sixteen patients (39%) had metastatic disease (M1). Median OS was 11.9 months for M0 cRT, 9.8 months for M0 pRT, and 2.8 months for M1 pRT (p = 0.003). The pRT provided clinical benefit in 72% of patients: the median OS for M0 patients who did and did not experience symptoms improvement from reirradiation was 9.8 and 5 months, respectively; M1 patients with and without therapeutic effect had a median OS of 4.5 and 2.2 months, respectively (p < 0.001). Acute toxicity was low, with 5 pRT patients requiring a nasogastric tube and 1 patient developing grade 3 skin toxicity. No grade ≥ 4 toxicities were reported.

**Conclusion:**

Palliative reirradiation in patients with recurrent esophageal cancer showed clinical benefit in 72% of the patients, with impact on OS both in M0 and M1 patients and low rate of acute toxicity. These findings support the use of reirradiation as a feasible and effective strategy in selected patients with recurrent esophageal cancer.

## Highlights

• According to our results, reirradiation for recurrent esophageal cancer appeared to be feasible and well tolerated.
• Clinical benefit was significantly associated with improved overall survival.
• Acute and late toxicity of treatment was low and manageable.

## Introduction

Esophageal cancer is the eighth most common cancer worldwide and the sixth leading cause of cancer-related mortality [1]. Treatment options for localized disease vary based on histology and tumor location. Both chemoradiation followed by surgery [2] and chemoradiation alone [[3], [4], [5]] are established as standard of care approaches. These treatment strategies are primarily used for squamous cell carcinoma, while perioperative chemotherapy is increasingly adopted for adenocarcinoma [6,7]. Chemoradiation remains the preferred option for patients who are unsuitable for surgery, regardless of tumor histology. Additionally, palliative chemotherapy is administered to patients with metastatic disease, and palliative radiotherapy is used to alleviate dysphagia in these patients. Despite advances in treatment, locoregional failure after definitive therapy remains the most common form of relapse, with 45–60% of patients experiencing local recurrence, depending on histology and initial stage of the disease [[8], [9], [10], [11]]. The estimated overall survival (OS) at 1 year after the diagnosis of local recurrence ranges from 70% to 97%, but these rates have a steep decline in the following months [[10], [11], [12]], independently from the treatment received.

Despite the high number of relapses, no standardized treatment approach has been established for managing local recurrence, and, to our knowledge, no study assessed the use of repeated irradiation for esophageal cancer in a western population. Only few retrospective studies focus on the treatment of recurrent esophageal cancer [[12], [13], [14], [15], [16], [17]] and they normally consider squamous cell carcinoma patients from Eastern countries, where the incidence of esophageal cancer is higher [1]. For patients in good clinical condition and whose primary treatment included chemoradiation (either as definitive or neoadjuvant therapy), salvage surgery and re-irradiation, with or without chemotherapy (CT), are potential treatments for recurrence [12,13,16,17]. Nonetheless, these interventions are associated with inherent limitations. Surgery includes a high incidence of adverse events such as pulmonary complications (17–30%), anastomotic leakage (17–39%), intensive care unit admission (17–22%), and postoperative mortality (3–15%) [10,12,13,17]. Reirradiation, on the other hand, faces limitations related to the cumulative radiation dose received by surrounding tissues and the risk of severe complications such as esophageal fistula and hemorrhage. [[10], [11], [12], [13],^17^].

When reirradiation is used for relapse, the median OS reported in literature is around 20 months if the treatment is administered with a curative intent [[12], [13], [14],^16^]. However, survival decreases dramatically during years, with OS rates dropping to 18–20% at 3 years, even when using high dose radiotherapy regimens combined with chemotherapy [11]. Conversely, robust data on palliative reirradiation are lacking because no existing study focuses on low dose retreatment. The only study focusing on a palliative subgroup of patients [15] reported different schedules of radiotherapy including high dose treatments, thus making the estimation of outcomes from palliative-dose reirradiation challenging.

Regarding treatment-related adverse events, most available data come from studies on repeated curative-dose radiotherapy. Esophageal fistula is reported in 9–20% of cases [10,12,13,17], with rates reaching 30% in a Korean population [18]. Radiation-induced pneumonia occurs in 12–24% of cases [10,12,17], although severe complications are relatively rare. Other adverse events, such as dysphagia and pericardial issues, are also common but typically not severe [12,17,18]. Currently, there is limited information on the toxicity of palliative reirradiation.

The aim of this study is to evaluate the survival outcomes and toxicity in patients treated with both palliative and curative reirradiation for esophageal cancer.

## Methods

### Patients

This study was approved by the institutional review board (ID MEC-2025–0061) of the Erasmus Medical Centre. Patients diagnosed with esophageal squamous cell carcinoma or adenocarcinoma who received a second course of radiotherapy for a local recurrence at the Radiotherapy Department of the Erasmus Medical Center between May 2015 and August 2024 were enrolled. Both patients with metastatic disease or locally advanced-only disease at initial diagnosis and/or at relapse were included. Clinical and pathological staging was performed according to the 8th edition of the American Joint Committee on Cancer TNM staging system based on available imaging for each patient (echo-endoscopy, CT, FDG-PET). Toxicities were scored using the Common Terminology Criteria for Adverse Events (CTCAE), version 5.0. Toxicity was classified as acute if it occurred within 3 months of reirradiation, and as late if it occurred after 3 months.

### Treatment

Palliative retreatment was delivered with the schedules of 20 Gy in 5 fractions (23.3 Gy EQD2_10_) or 30 Gy in 10 fractions (32.5 Gy EQD2_10_). For patients who received reirradiation with curative intent, the total dose was 50.4 Gy delivered in 28 fractions (49.6 Gy EQD2_10_). Chemotherapy was given concurrently with radiotherapy when the treatment intent was curative and it consisted of weekly carboplatin-paclitaxel as per CROSS regimen [2]. The doses delivered were recalculated for homogeneity among the different schedules adopted, using the equivalent dose formula: EQD2 = D x [d + α/β)/(2 + α/β), where D is the total dose delivered, d the dose per fraction, considering an α/β of 10 (EQD2_10_). The treatment schedules of the first treatment are shown in table 1.

### Follow- up and statistical analysis

The follow-up was conducted according to standard clinical practice. At relapse after the first irradiation, local recurrence was defined as esophageal or anastomotic recurrence or lymph node metastases including the supraclavicular level. These recurrences were classified as follows: primary failure if localized to the esophagus/anastomosis; nodal failure if isolated nodal relapse limited to regional lymph nodes; and metastatic failure if metastases were found in other organs or non-regional lymph nodes in addition to primary or isolated nodal recurrence.

After reirradiation, local failure was defined as the persistence or worsening of local symptoms after 4 weeks from the reirradiation, and/or tumor progression diagnosed with endoscopic ultrasound, CT scan or PET scan. Local control (LC) was calculated as the interval between the first day of reirradiation until the event of local failure or last follow-up. Overall survival (OS) was calculated from the first day of reirradiation to death or last follow-up.

The clinical benefit of reirradiation was assessed 3–4 weeks after the final radiation fraction, defined as a reduction of dysphagia, pain, or bleeding, compared to baseline symptoms. Patients with a stent placed before the reirradiation were considered as not evaluable for dysphagia at baseline and were also not scored as an adverse event from the treatment.

OS and LC were calculated with the Kaplan-Meier method. A P-value of < 0.05 was considered statistically significant. For the analysis, different subgroups of patients were defined, considering the metastatic status: non metastatic patients (M0) if without evidence of distant metastases and metastatic patients (M1) if distant metastases in addition to the esophageal relapse were present. Moreover, patients were also classified based on the intent of reirradiation (curative RT = cRT, palliative RT = pRT) and the clinical benefit from treatment, that was defined as in improvement in pain, dysphagia or bleeding within 4 weeks from the reirradiation. Given the small number of patients, no matched cohort analysis considering confounding factors (such as histology, performance status or age) was made, but only a descriptive analysis on patterns of relapse.

## Results

### Patient characteristics

Forty-one patients with locally recurrent esophageal cancer were included in the study. Most patients had a histologically confirmed diagnosis of adenocarcinoma (n = 37, 90%), while10% (n = 4) of squamous cell carcinoma. Most tumors were located in the distal esophagus (88%, n = 36), while only 2 and 3 cases were located in proximal and middle esophagus, respectively. Patient characteristics and treatment details are shown in Table 1.

**Table 1:** Patients and treatment characteristics.

Patient and treatment characteristics (N = 41)	N (%) or median (IQR)
**Age at reirradiation**	73.5 (55–88.1)
**Gender**	
Male	34 (83%)
Female	7 (17%)
**Histology**	
Adenocarcinoma	37 (90%)
Squamous Cell Carcinoma	4 (10%)
**Localization of primary tumor**	
Proximal	2 (5%)
Middle	3 (7%)
Distal	36 (88%)
**Stage at diagnosis of primary tumor**	
IIb	4 (10%)
III	23 (56%)
IVa	2 (5%)
IVb	12 (29%)
**Type of relapse**	
Primary failure	21 (52%)
Lymph node failure	5 (12%)
Both primary and lymph node	10 (24%)
Primary, lymph node and metastatic	5 (12%)
**Relapse free interval**	10.2 (0.7–38)
**Initial radiotherapy schedule**	
50.4 Gy in 28 fractions (49.6 Gy EQD2_10_) (+CTx)	2 (5%)
41.4 Gy in 23 fractions (40.7 Gy EQD2_10_) (+CTx)	16 (39%)
30 Gy in 10 fractions (23.3 Gy EQD2_10_)	10 (24%)
20 Gy in 5 fractions (23.3 Gy EQD2_10_)	13 (32%)
**Reirradiation schedules**	
50.4 Gy in 28 fractions (49.6 Gy EQD2_10_)	5 (12%)
30 Gy in 10 fractions (32.5 Gy EQD2_10_)	4 (10%)
20 Gy in 5 fractions (23.3 Gy EQD2_10_)	32 (78%)
**Cumulative dose (EQD2_10_)**	55.8 (35.0–90.3)

Abbreviations: N = number; IQR = inter quartile range; Gy = Gray; EQD2_10_ = Equivalent dose of 2 Gy per fraction with a/b ratio of 10 Gy; CTx = chemotherapy.

Twelve patients (29%) had metastatic disease at the time of first diagnosis, and 5 patients (12%) were found to have distant disease at relapse (in 3 cases associated with both primary and nodal failure, in 2 cases with only primary failure). Overall, at the time of reirradiation, 16 patients (39%) had metastases (M1) and 25 patients (61%) had no metastases (M0). Among patients with no metastatic relapse, 21 (52%) patients presented a primary failure and 5 (12%) a lymph node failure; ten patients (24%) experienced both primary and lymph node relapse. Primary failure was mainly observed both in proximal and distal locations, while tumors of the middle esophagus relapsed with lymph node only recurrence. Furthermore, adenocarcinoma relapsed with primary failure in more than a half of cases (54%, n = 20). Detailed patterns of relapse are shown in table 2. All metastatic patients were re-treated with low dose palliative radiotherapy with a median total dose of 20 Gy (range 8–30 Gy; equal to 23.3 Gy EQD2_10_). Of the 25 patients without metastases, 20 (80%) were treated with palliative radiotherapy (median dose 20 Gy, range 20–30 Gy; equal to 23.3 Gy EQD2_10_) while the remaining 5 non-metastatic patients (20%) were treated with definitive chemoradiotherapy (50.4 Gy in 28 fractions; equal to 49.6 EQD2_10_; chemotherapy: carboplatin and paclitaxel). Among the 5 patients receiving reirradiation with a curative dose, only one presented with complete overlap of previously irradiated area. Two patients presented with a marginal overlap of fields while the other two patients had no overlap at all. All the palliative-treated patients had complete overlap of irradiated fields between the two courses of radiotherapy.

The median age at reirradiation was 74 years (range 55–88 years). The median relapse-free interval after the first treatment was 10.2 months (range 0.7–38 months), while the median follow-up after the second radiotherapy was 5 months (range 0.9–61.8 months). At reirradiation, most patients presented with mild to moderate symptoms. In particular, 71% (n = 29) and 7% (n = 3) of patients presented with G1–2 and G3 dysphagia, respectively; furthermore, 7 patients had a nasogastric tube placed before the start of reirradiation due to severe dysphagia caused by disease progression. Two patients (5%) had no swallowing difficulties. Pain was less frequent at baseline, as 68% (n = 28) of patients reported no pain and 32% (n = 13) had G1–2 pain, with no cases of severe pain. Bleeding was absent in the majority of patients (81%, n = 33), though 12% (n = 5) experienced G1–2 and 3 patients (7%) G3 bleeding. Baseline symptoms are further detailed in Table 3. As initial treatment, 18 patients (44%) received concurrent chemoradiotherapy, while the remaining 23 patients received palliative radiotherapy. The median delivered dose was 32.5 Gy EQD2_10_ (range 23.3–49.6 Gy EQD2_10_). The median cumulative dose of first and second course of radiotherapy was 56 Gy EQD2_10_.

### Outcomes

**Fig. 1:**
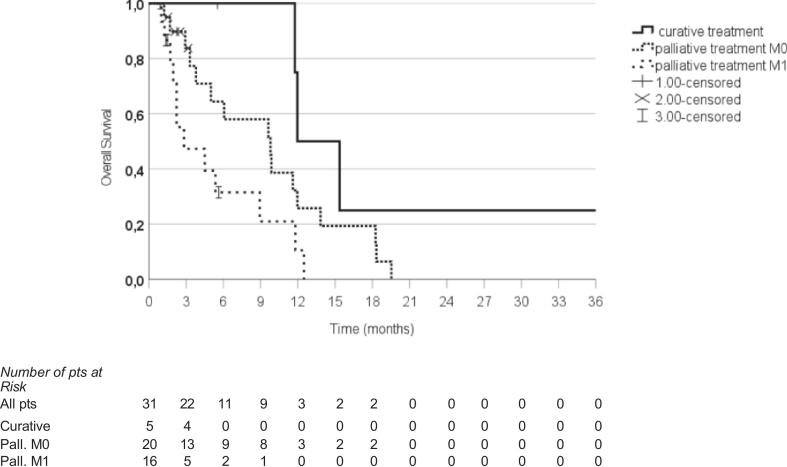
Abbreviations: M0 = non metastatic patients; M1 = metastatic patients; pts = patients.

The median OS for the entire cohort was 9.6 months: 31 patients died during follow-up. Considering the palliative reirradiation setting, the median OS for M0 patients was 9.8 months, while for M1 patients this was 2.8 months (p = 0.037). For curative radiotherapy, the median OS was 11.9 months. OS in patients treated with curative intent was significantly higher than in patient treated with palliative intent, both with M0 and M1 disease (p = 0.003, Fig. 1). The estimated 6-month OS rates for M0-cRT, M0-pRT, and M1-pRT were 75%, 58%, and 21%, respectively, while at 1 year, the rates were 25%, 19%, and 0%, respectively.

**Fig. 2:**
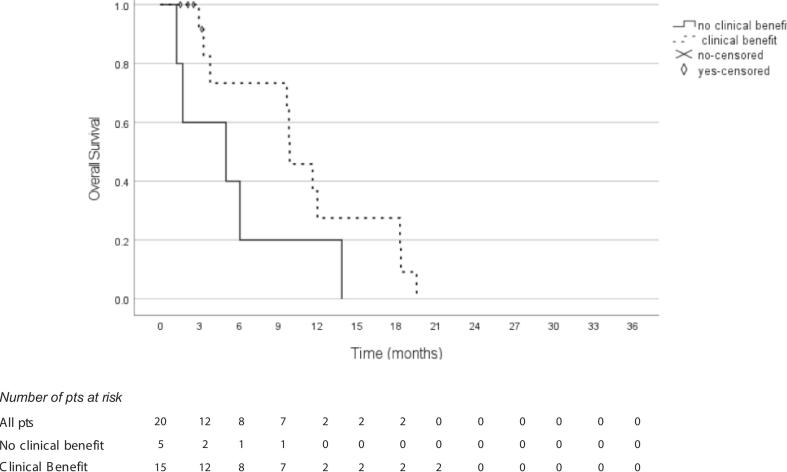
Abbreviations: M0 = non metastatic; pts = patients.

Considering p-RT only, 26 out of 36 patients (72%) had a clinical benefit from the treatment. 12 patients had a reduction of dysphagia, 4 patients a reduction of the pain, 3 patients a reduction of the bleeding. 7 patients had a combined effect: a reduction of dysphagia and pain was seen in 3 patients, and 4 patients had a reduction in dysphagia and bleeding. At subgroup analysis, M0 patients with a clinical benefit had a median OS of 9,8 months compared to a median OS of 5 months for M0 patients without clinical benefit (p < 0.001), (Fig. 2).

Focusing on the p-RT M1 subgroup, 11 out of 16 patients (69%) had an improvement of symptoms after palliative reirradiation. Moreover, M1 patients with clinical benefit had a median OS of 4.5 months while M1 patients without clinical gain had a median OS of 2.2 months (p < 0.001).

The actuarial LC in the overall population at 6 months was 60% and 32% at 1 year. The median local recurrence free interval was 9.6 months. Furthermore, when patients experienced a clinical benefit, the LC was significantly better in comparison to patients without symptoms improvement (12.9 versus 2.3 months, p 0.02).

### Toxicity

No grade ≥ 4 acute toxicity was reported. In total, 7 patients (15%) had a grade 3 acute toxicity. During the month after reirradiation, 5 patients experienced a worsening of dysphagia and required a nasogastric tube. One patient was admitted during the radiotherapy course due to shortness of breath and general deterioration. As no cause of these symptoms was found, it was scored as grade 3 acute side effect of the radiotherapy. Lastly, 1 patient had an extensive desquamation of the skin in the neck. Symptoms at baseline and acute toxicity are shown in Table 2. Seven patients had a nasogastric tube placed before the start of radiotherapy: for this reason, they were considered as not evaluable for dysphagia at baseline and as an adverse event from radiotherapy. Furthermore, in 3 patients a stent was placed before reirradiation, and they were treated with the stent in place. No patients required invasive nutritional support, such as a percutaneous endoscopic gastrostomy (PEG) or parenteral nutrition. Moreover, no grade ≥3 late toxicities were reported.

**Table 2:** Patterns of relapse according to baseline localization and histology.

	Primary faiulure N (%**)	Lymph node failure N (%**)	Primary and lymph node failure N (%**)	Primary + lymph node + metastatic failure (N%**)
Localization (N, %*)				
Proximal esophagus (N = 2, 5%)	2 (100%)	0 (0%)	0 (0%)	0 (0%)
Middle esophagus (N = 3, 7%)	0 (0%)	2 (67%)	1 (33%)	0 (0%)
Distal esophagus (N = 36, 88%)	19 (53%)	3 (8%)	9 (25%)	5 (14%)
Histology (N, %*)				
Squamous cell carcinoma (N = 4, 10%)	1 (25%)	2 (50%)	1 (25%)	0 (0%)
Adenocarcinoma (N = 37, 90%)	20 (54%)	3 (8%)	9 (24%)	5 (14%)

* % out of all the 41 patients; ** % out of the number of patients in the specific subgroup.

**Table 3:** Symptoms related to reirradiation.

Symptom, grade	Baseline, N (%)	Acute, N (%)
**Dysphagia**		
G 0	2 (5%)	8 (19%)
G 1–2	29 (71%)	20 (50%)
G 3	3 (7%)	5 (12%)
Not evaluable	7 (17%)	8 (19%)
**Pain**		
G 0	28 (68%)	27 (66%)
G 1–2	13 (32%)	14 (34%)
G 3	0	0
**Bleeding**		
G 0	33 (81%)	39 (95%)
G 1–2	5 (12%)	2 (5%)
G 3	3 (7%)	0
**Skin toxicity**		
G 0	0 (100%)	38 (93%)
G 1–2	−	2 (5%)
G 3	−	1 (2%)
**Nausea**		
G 0	0 (100%)	31 (76%)
G 1–2	−	10 (24%)
G 3	−	0
**Vomiting**		
G 0	0 (100%)	35 (85%)
G 1–2	−	6 (15%)
G 3	−	0

Abbreviations: N = number of patients; G = grade according to CTCAE v.5; Not evaluable: a nasogastric tube is present at start of reirradiation; progressive disease within 1 month from the start of reirradiation or stent placement due to progressive disease and not due to toxicity.

## Discussion

In our trial, we found a significantly higher median OS of 11.9 months for non-metastatic patients who underwent reirradiation with a curative intent, compared to 9.8 months for non-metastatic patients treated with palliative reirradiation. In contrast, patients with distant metastases and treated with palliative reirradiation had a significantly shorter OS of 2.8 months (p 0.03).

To our knowledge, no other trial has specifically addressed the efficacy of repeated palliative reirradiation, making comparison with current literature challenging. Some studies on reirradiation after a curative treatment include data on low-dose regimens, but they don’t differentiate outcomes based on the radiation schedule. Moreover, the number of palliative-treated patients is low, thus making the results of palliative radiotherapy not robust enough. Xiang et al. reported the number of patients receiving palliative reirradiation in their trial: only 4 out of 30 patients received a reirradiation dose of <40 Gy [16]. Unfortunately, the reported median OS of 34.5 months in this study is cumulative and not differentiated by dose, leading to overestimation as more than 60% of patients received a curative dose.

We found a median OS of 2.8 months in the 16 patients treated with a palliative dose who had metastatic disease which is lower than the OS of 6.5 months found in the study of Yamaguchi et al. [15]. They conducted a retrospective study in which patients were classified as palliative if they had an ECOG performance status >2, dysphagia >G1, and metastatic disease. Patients received doses ranging from 16 to 60 Gy, with a median dose of 36 Gy. The difference in OS may be due to the higher reirradiation doses used in their study, however the exact number of patients who received a palliative-dose is not reported. Furthermore, literature about survival in best supportive care cohorts report OS rates ranging from 8 to 14 months [12,13,17], but these studies typically involve locoregional-only recurrence, which may overestimate survival as they include both metastatic and non-metastatic palliative patients. So, no direct comparison can be made between patients treated with palliative reirradiation and those managed with best supportive care.

When considering reirradiation with a curative-dose, our observed median OS of 11.9 months is lower than the 17–34 months reported in other studies [12,13,14,15,16]. Notably, the highest median OS of 34 months was reported by Xiang et al. [16], who treated 60% of the patients with a dose of ≥50 Gy and a maximum dose of 63 Gy. Similarly, Hong et al. [12] reported a median OS of 21 months in 20 patients who received up to 70 Gy. In both studies, the median dose of initial RT was 60 Gy, which is higher than the dose used in our cohort. Moreover, the median follow-up of the study of Hong was 87 months, while this in the study of Xiang was not reported. The difference in overall survival (OS) may be attributed to the small number of patients (n = 5) in our cohort who received repeated curative radiotherapy, which may not be fully representative. Also, the doses used in both treatment courses in our study were lower compared to those in literature. However, 2 out of 5 patients were alive at the last follow-up, suggesting that the true survival benefit may be underestimated. The limitations regarding the curative reirradiation in our study limit the the generalizability of survival outcomes in this subgroup.

Clinical benefit after reirradiation was reported in 11 out of 16 metastatic patients, highlighting its role in symptoms management for this group. Symptoms improvement is an endpoint that is rarely considered in studies of esophageal reirradiation. We found that 72% of palliative patients experienced clinical benefit, with notable symptom improvement even among M1 patients. Furthermore, the clinical benefit was correlated with an improvement in OS both for M0 and M1 palliative-treated patients (9.8 vs 5.0 months for M0 and 4.5 vs 2.2 months for M1, for clinical benefit vs no clinical benefit). The observed clinical benefit may be related to the low incidence of adverse events in our study. No acute or late grade ≥4 toxicities were reported, and only 5 patients required a nasogastric tube for grade 3 dysphagia during the second RT course. This low incidence of severe adverse events may be attributed to the relatively low cumulative doses received by more than half of the patients, who had already received palliative doses during the first irradiation course.

The retrospective nature of the study and the heterogeneous population of patients obviously limits the generalizability of our results. In particular, the outcomes of the curative reirradiation cohort could be not fully representative, given the small number of patients considered in this subgroup. Furthermore, about the palliative reirradiation group, no specific information about indications for reirradiation based on histology or esophageal localization could be addressed, due to the small number of patients considered in each subgroup.

Despite this, considering the improvement in symptoms burden and in OS, and the low rate of toxicity, our results may be useful in the clinical decision-making during the multidisciplinary tumor board, especially for patients with limited therapeutic options or in a palliative setting.

## Conclusions

In our study, 72% of palliative-treated patients derived a clinical benefit from the treatment, even if they had metastasis at the beginning of reirradiation. When considering M0 patients treated with palliative radiotherapy, those who had a clinical benefit had a median OS of 9,8 months, compared to a median OS of 5 months for M0 patients without clinical benefit (p < 0.001). Furthermore, acute and late toxicity was low and manageable. This makes reirradiation a valid treatment option, particularly in the palliative setting,

## Declaration of Competing Interest

The authors declare the following financial interests/personal relationships which may be considered as potential competing interests: Joos J. Nuyttens reports a payment to institution from Accuray Inc for a presentation and is a board member of AEX board. Lucy A. Werkhoven reports a payment to institution from Accuray Inc for a presentation. Chiara Mattioli reported no conflict of interest. Mauro Loi reported no conflict of interest.
